# Calling an ambulance for non‐emergency medical situations: Results of a cross‐sectional online survey from an Australian nationally representative sample

**DOI:** 10.1111/1742-6723.14086

**Published:** 2022-09-16

**Authors:** Brennen Mills, Michella Hill, Alecka Miles, Erin Smith, Eben Afrifa‐Yamoah, David Reid, Shane Rogers, Moira Sim

**Affiliations:** ^1^ School of Medical and Health Sciences Edith Cowan University Perth Western Australia Australia; ^2^ School of Science Edith Cowan University Perth Western Australia Australia; ^3^ School of Arts and Humanities Edith Cowan University Perth Western Australia Australia

**Keywords:** ambulance, comprehension, emergency, perception, service utilisation

## Abstract

**Objective:**

To investigate the Australian general public's perception of appropriate medical scenarios that warrants a call to an emergency ambulance.

**Methods:**

An online survey asked participants to identify the likely medical treatment pathway they would take for 17 hypothetical medical scenarios. The number and type of non‐emergency scenarios (*n* = 8) participants incorrectly suggested were appropriate to place a call for an emergency ambulance were calculated. Participants included Australian residents (aged >18 years) who had never worked as an Australian registered medical doctor, nurse or paramedic.

**Results:**

From a sample of 5264 participants, 40% suggested calling an emergency ambulance for a woman in routine labour was appropriate. Other medical scenarios which were most suggested by participants to warrant an emergency ambulance call was ‘Lego in ear canal’ (11%), ‘Older person bruising’ (8%) and ‘Flu’ (7%). Women, people aged 56+ years, those without a university qualification, with lower household income and with lower emotional wellbeing were more likely to suggest calling an emergency ambulance was appropriate for non‐emergency scenarios.

**Conclusions:**

Although emergency healthcare system (EHS) capacity not increasing at the same rate as demand is the biggest contributor to EHS burden, non‐urgent medical situations for which other low‐acuity healthcare pathways may be appropriate does play a small role in adding to the overburdening of the EHS. This present study outlines a series of complaints and demographic characteristics that would benefit from targeted educational interventions that may aid in alleviating ambulance service attendances to low‐acuity callouts.

## Graphical abstract

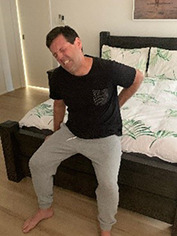

**Key findings**

- Calls for emergency ambulance utilisation for non‐emergency conditions contributes to the overburdening of emergency healthcare systems.
- Women, older people (56+ years), those without a university qualification, lower household income and lower emotional wellbeing were factors increasing the likelihood of suggesting a call would be placed for an emergency ambulance for non‐emergency situations.
- A woman going through routine labour was by far the non‐emergency scenario members of the general public most frequently suggested warranted engagement with emergency ambulance services.

## Introduction

Ambulance demand continues to rise at a rate higher than population growth. Between 2008 and 2015 in Victoria, Australia, ambulance demand rose by 29.2%.^1^ Increased ambulance utilisation contributes towards ED overcrowding, ambulance ramping and lowered access to care and ambulances in the community,^2^ each with the capacity to lead to diminished patient outcomes.^3^ These factors place substantial and ongoing burden on emergency healthcare workers.

Increased demands on emergency healthcare services (EHS) have been attributed to a growing elderly population and increasing population co‐morbidities,^4^ along with public hospital acute bed capacity not increasing at the same level as demand.^5^ Although evidence suggests Australians can appropriately self‐refer to the ED,^6^ and overcrowding is overwhelmingly attributable to a lack of system capacity to meet increasing demand,^7^ non‐urgent presentations that could be effectively managed via alternate primary healthcare pathways can divert EHS resources from patients with serious/acute medical emergencies.^8^, ^9^ Cases attended by Victorian paramedics requiring no intervention from paramedics increased by on average 6.7% annually between 2008 and 2015.^1^ Further, 21.2% of secondary telephone triage cases between 2009 and 2012 were considered not suitable for transport to ED.^10^

Patients frequently perceive urgency of their medical conditions to be greater in comparison to medical practitioners.^11^, ^12^ The present research aims to extend upon a previous investigation^13^ exploring public understanding of appropriate medical response to non‐emergency situations not requiring immediate emergency intervention, whereby a call placed to Triple Zero (000) for an emergency ambulance would be unwarranted.

## Methods

### Study design

Cross‐sectional via online survey.

### Participants

Prospective participants included any Australian resident aged >18 years who was not currently nor had ever before worked as an Australian registered medical doctor, nurse or paramedic. Participants were recruited through an online market research company *Pureprofile*.

### Ethics approval

Ethics approval was granted by the Edith Cowan University Human Research Ethics Committee (#2020‐01958).

### Materials

#### Medical scenarios

Participants were presented with 17 hypothetical medical scenarios (Table 1) and asked what healthcare pathway (from a list of nine options; Table 2) would they likely undertake if presented with this scenario in real life. Among these response options was an ‘Other’ option allowing participants to type in open‐ended responses if they felt their response would differ from one of the eight prompted response options. Participants could only choose one response option. Open‐ended responses were coded as ‘Call 000 for an Ambulance’ if participants indicated they would call for an ambulance in the first instance. All other legible responses were coded as not calling 000 for an Ambulance.

**TABLE 1:** Non‐emergency (emergency ambulance utilisation *not* recommended) and emergency (emergency ambulance utilisation recommended) scenario text presented to participants

Scenario number	Short scenario title	Full scenario text
Non‐emergency scenarios		
2	Flu	A 45‐year‐old male has flu‐like symptoms. He has a mild fever, cough, headache, runny nose and feels tired.
4	Older person bruising	A 77‐year‐old woman knocks herself against the kitchen table, and a large bruise immediately appears on her thigh.
6	Lego in ear canal	A 4‐year‐old girl has a Lego piece stuck in her ear canal.
7	Stubbed toe	A 25‐year‐old male is playing football with his friends in his backyard with his bare feet. He stubs his toe on a brick. There is blood and he suggests it is throbbing quite painfully.
10	Alcohol intoxication	A 22‐year‐old male is conscious, not injured and has drunk a substantial amount of alcohol on a night out.
11	Woman in labour	A 33‐year‐old woman is 9 months pregnant and goes into early stages of labour. Her waters have broken, and she feels uncomfortable.
12	Back pain	A 40‐year‐old man with a 6‐month history of back pain wakes up in the middle of the night with a sore back and has run out of pain killers. The man is in quite a bit of pain.
14	Cut finger	A 42‐year‐old man has cut his finger while chopping vegetables, and the bleeding is controlled with pressure.
Emergency scenarios		
1	Box Jellyfish sting	While in Northern Queensland, a boy is stung by a Jellyfish while swimming at the beach, and large welts appear on his arm.
3	Snake bite (unidentified)	A 50‐year‐old woman has been bitten on her ankle by an unidentifiable snake.
5	Mild chest pain	A 40‐year‐old woman is experiencing mild chest pain. She does not think it is indigestion or a strained muscle.
8	Stroke	A 67‐year‐old man is slurring his words; he has not drunk any alcohol.
9	Severe chest pain	A 52‐year‐old man has severe chest pain, is sweating and grey in colour.
13	Paracetamol overdose	A 32‐year‐old female has taken 10 regular paracetamol tablets in the last 12 h, and is feeling extremely unwell. She has abdominal pain and feels nauseous.
15	Child head haematoma	A 3‐year‐old boy has fallen off the couch and bumped his head. He began crying immediately and a golf‐ball size lump with a bruise promptly appears.
16	Potential meningococcal	A 4‐year‐old girl has woken up with a high temperature, feels hot to touch, has a really sore neck and a headache which Panadol is not relieving.
17	Older person hip pain	A 80‐year‐old woman feel out of bed, is now unable to get up and is complaining of hip pain on her right side.

**TABLE 2:** Scenario response options provided to participants

Call 000 for an Ambulance
Go to the ED
Make an appointment to visit a GP
Talk to a pharmacist
Make an appointment at a COVID clinic
Call Healthdirect or Nurse‐On‐Call
Provide first aid
No immediate action but monitor situation
Other

The 17 hypothetical scenarios were re‐purposed from a previous investigation^13^ exploring the general public's ability to correctly categorise emergency *vs* non‐emergency medical scenarios. A panel of experienced registered paramedics (*n* = 5) reached 100% consensus on their interpretation of whether medical scenarios were of sufficient risk or severity to warrant an emergency call to 000 for an ambulance or not. This process involved the panel meeting with members of the research team as a group, and was presented with each of the 17 scenarios. Following presentation of a scenario, panel members were asked to (confidentially) write down whether they felt the scenario should or should not warrant a call for an emergency ambulance. Where responses were not 100% unanimous across the panel, research team members were to facilitate discussion until consensus was reached. Of the 17 scenarios, nine were identified as emergencies warranting a call to 000 for an ambulance with the remaining eight deemed as non‐emergencies. For the present research study, the nine ‘emergency’ scenarios were hidden among ‘non‐emergency’ scenarios and displayed to study participants as ‘red herrings’ to make distinguishing the non‐emergency medical scenarios less obvious. Scenario display order was universal across study participants; however, all 17 scenarios were randomly mixed to provide a finalised scenario display order.

Given incorporation of images alongside text can improve comprehension of information being presented, particularly among people with low literacy skills,^14^ graphical depictions of medical scenarios were provided to complement scenario text (images provided as Appendix S1). Photos were either original creations taken with a 12‐megapixel wide‐angle camera found on an Apple iPhone X (Cupertino, CA, USA), or sourced from stock photos (Dreamstime.com).

#### Demographics

In the online questionnaire participants initially completed a demographics section. The demographics section included questions on age, sex, identification as Aboriginal or Torres Strait Islander, highest level of education, metropolitan or rural residence, employment, annual household income, number of children and whether or not they suffered from any chronic illness or had a disability. Participants were also asked to complete the Brief Emotional Experience Scale (BEES) as a measure of emotional wellbeing. The BEES comprises of three positive (Happy, Calm, Confident) and three negative (Worried, Sad, Afraid) emotional adjectives rated on a 4‐point response scale: (1) Not at all; (2) A little bit; (3) Quite a bit; and (4) A lot. An overall emotional wellbeing score is calculated by summing across the positive and negative adjectives separately, and then subtracting the negative score away from the positive score. The overall score can range from −9 to +9 where a higher score indicates greater self‐reported emotional wellbeing.

### Procedure

Participants were sent an invitation to participate in the research through their online *Pureprofile* account. The online survey was active from 19 November 2019 to 2 December 2020, facilitated through the *Qualtrics* survey platform. Upon completion of the survey, *Pureprofile* facilitated financial reimbursement for participant's time. Estimated time to complete the survey was 20 min.

### Analysis

For the eight non‐emergency scenarios, participants were coded as either (incorrectly) calling 000 for an ambulance or choosing any other healthcare pathway. The number of non‐emergency scenarios participants incorrectly coded as warranting a call to 000 for an ambulance was calculated (scored out of 8). Descriptive statistics were calculated and significant differences within groups determined using *t*‐tests and one‐way ANOVAs. Generalised linear modelling assuming binomial distribution was used to study the relationships between key demographic variables and the number of non‐emergency scenarios correctly identified as not warranting a call to 000 for an ambulance.

## Results

A total of 6723 individuals began the online survey. Of these, 109 participants did not proceed passed the first page containing a detailed participant information letter. A further 30 participants were screened out for identifying as under 18 years of age, a further 112 for not being an Australian resident, and a further 752 for suggesting they had previously worked in Australia as a registered doctor, nurse or paramedic. Last, a total of 89 participants were further screened out as they completed demographic information only. This left a final sample of 5631 eligible participants. A total of 5264 participants completed all 17 scenarios. Given no significant differences were noted across any demographic factors (e.g., age, sex, income, BEES score) for those who did and did not provide responses to all 17 medical scenarios, missing data was deemed missing completely at random. Demographics for the final sample are outlined in Table 3. Missing data was associated with some demographic variables where participants chose not to disclose information. These are not reflected in Table 1. These include sex *n* = 14 (0.27%); Aboriginal/Torres Strait Islander status *n* = 37 (0.70%); relationship status *n* = 203 (3.86%) and household income *n* = 517 (9.82%).

**TABLE 3:** Final sample demographics with number of non‐emergency scenarios (out of 8) incorrectly suggesting they would call an emergency ambulance for

Demographics	*N* (%)	Non‐emergency scenarios	
		Mean (SD)	*P*‐value
Sex			<0.001*
Male	2232 (42.5)	1.04 (1.35)	
Female	3018 (57.5)	0.71 (1.11)	
Age			<0.001*
18–35	1415 (26.9)	0.71 (1.25)	
36–55	1725 (32.8)	0.78 (1.28)	
56+	2124 (40.3)	1.01 (1.16)	
Aboriginal/Torres Strait			0.766
Yes	162 (3.1)	0.82 (1.18)	
No	5065 (96.2)	0.85 (1.23)	
Residency			0.015*
Metropolitan	4088 (77.7)	0.83 (1.20)	
Regional	1176 (22.3)	0.93 (1.33)	
Relationship status			<0.001*
Married	2725 (51.8)	0.84 (1.19)	
*De facto*	702 (13.3)	0.65 (1.15)	
Single	1634 (31.0)	0.93 (1.33)	
Level of education			<0.001*
Did not graduate high school	470 (8.9)	1.07 (1.41)	
High school	1069 (20.3)	0.96 (1.38)	
Trade or TAFE	1548 (29.4)	0.86 (1.17)	
Undergraduate	1391 (26.4)	0.76 (1.19)	
Postgraduate	786 (14.9)	0.71 (1.06)	
Income earner			<0.001*
Yes	2949 (59.4)	0.76 (1.21)	
No	2014 (40.6)	0.96 (1.25)	
Income			<0.001*
$1–$10 399	103 (2.3)	1.36 (1.84)	
$10 400–$15 599	65 (1.5)	1.14 (1.59)	
$15 600–$20 799	125 (2.7)	1.01 (1.27)	
$20 800–$31 199	408 (8.9)	1.11 (1.47)	
$31 200–$41 599	366 (8.0)	0.96 (1.11)	
$41 600–$51 999	435 (9.5)	1.05 (1.44)	
$52 000–$64 999	431 (9.4)	0.85 (1.12)	
$65 000–$77 999	418 (9.1)	0.88 (1.25)	
$78 000–$103 999	764 (16.7)	0.71 (1.07)	
$104 000+	1456 (31.9)	0.64 (1.07)	
Children under 18			<0.001*
Yes	1486 (29.4)	0.73 (1.30)	
No	3575 (70.6)	0.89 (1.20)	
Chronic condition			0.060
Yes	1756 (34.7)	0.89 (1.24)	
No	3305 (65.3)	0.82 (1.23)	
BEES – total score			0.670
Positive score	2996 (59.2)	0.83 (1.15)	
Zero score	1147 (22.6)	0.86 (1.39)	
Negative score	918 (18.2)	0.86 (1.29)	
Disability			<0.001*
Yes	629 (12.6)	1.03 (1.46)	
No	4372 (87.4)	0.81 (1.18)	

^*^ Significant association at 5% level of significance.

### Suggesting non‐emergency scenarios warrant a call to 000

The mean score (out of 8) for the number of non‐emergency scenarios for which participants incorrectly suggested a call to 000 for an emergency ambulance was 0.84 (SD = 1.23). By far the most common non‐emergency scenario for which participants suggested an emergency ambulance was warranted was the ‘Woman in labour’ scenario (40.6% suggesting they would call an ambulance), followed by ‘Lego in ear canal’ (10.5%), ‘Older person bruising’ (7.5%) and ‘Flu’ (7.3%) (Table 4).

**TABLE 4:** Number and proportion of sample incorrectly calling for an emergency ambulance for different non‐emergency scenarios

Scenario number	Non‐emergent scenario	Number (%) incorrectly classified as an emergency
2	Flu	386 (7.3%)
4	Older person bruising	394 (7.5%)
6	Lego in ear canal	552 (10.5%)
7	Stubbed toe	293 (5.6%)
10	Alcohol intoxication	239 (4.5%)
11	Woman in labour	2136 (40.6%)
12	Back pain	313 (5.9%)
14	Cut finger	166 (3.2%)

### Demographic factors influencing likelihood of calling an emergency ambulance for non‐emergency scenarios

Table 5 depicts the results of the multivariate analysis demonstrating the impact of demographic variables on suggestions of calling 000 for an ambulance for non‐emergency scenarios. Compared to males, females were 33% less likely to call for an emergency ambulance for non‐emergency scenarios. Similarly, those aged 18–35 years were 21% less likely to call for an emergency for ambulance for non‐emergency scenarios compared to those aged 56+ years.

**TABLE 5:** Results of multivariate generalised linear equation model predicting demographic factors contribution to number of non‐emergency scenarios for which ‘Call 000 for an ambulance’ was the chosen healthcare pathway

Demographics	Estimate (SE)	AOR (95% CI)	*P*‐value
Sex			
Male	0	1	
Female	−0.3982 (0.0345)	0.6720 (0.6280, 0.7180)	<0.0001*
Age			
18–35	0	1	
36–55	0.0853 (0.0474)	1.0890 (0.9930, 1.1950)	*0.0721*
56+	0.1891 (0.0534)	1.2080 (1.0880, 1.3420)	0.0004*
Aboriginal/Torres Strait			
Yes	0	1	
No	0.0623 (0.0998)	1.0640 (0.8790, 1.3000)	0.5327
Residency			
Metropolitan	0	1	
Regional	0.0352 (0.0400)	1.0360 (0.9570, 1.1200)	0.3796
Relationship status			
Married	0	1	
*De facto*	−0.2416 (0.0598)	0.7850 (0.6980, 0.8820)	<0.0001*
Single	0.0969 (0.0411)	1.1020 (1.0160, 1.1940)	0.0185*
Completed university			
Yes	0	1	
No	0.1229 (0.0370)	1.1310 (1.0520, 1.2160)	0.0009*
Income earner			
Yes	0	1	
No	0.0361 (0.0407)	1.0370 (0.9570, 1.1230)	0.3750
Income			
$1–$41 599	0	1	
$41 600–$77 999	−0.0654 (0.0482)	0.9370 (0.8520, 1.0300)	0.1753
$78 000–$103 999	−0.2668 (0.0613)	0.7660 (0.6790, 0.8630)	<0.0001*
$104 000+	−0.2458 (0.0517)	0.7820 (0.7070, 0.8660)	<0.0001*
Children under 18			
Yes	0	1	
No	−0.0010 (0.0465)	0.9990 (0.9120, 1.0950)	0.9825
Chronic condition			
Yes	0	1	
No	0.0670 (0.0396)	1.0690 (0.9900, 1.1560)	*0.0907*
BEES – total score			
Positive score	0	1	
Zero score	0.1082 (0.0420)	1.1140 (1.0260, 1.2100)	0.0101*
Negative score	0.1074 (0.0472)	1.1130 (1.0140, 1.2210)	0.0229*
Disability			
Yes	0	1	
No	−0.0825 (0.0537)	0.9210 (0.8290, 1.0230)	0.1242

^*^ Significant association at 5% level of significance. *P*‐values in italics denote a trend towards significance.

Those without a university degree were 13% more likely to call for an emergency ambulance for non‐emergency scenarios, and there was a decreasing trend in the likelihood to call an emergency ambulance for non‐emergency scenarios for income; higher earning individuals were less likely to suggest they would call for an ambulance than lower income individuals. Further, those with negative mental health scores measured via the BEES were 11% more likely to call for an ambulance for non‐emergency scenarios than those with positive mental health scores.

## Discussion

### Summary of findings

Risk aversion is common when it comes to personal health, with a preference to request emergency medical intervention and not need it than require emergency medical intervention and not have it.^15^ Whereas this should not necessarily change, an appropriate balance needs to be found between risk aversion and overreliance/overburdening already stretched EHS.

Our findings are not the first to suggest members of the general public can miscategorise non‐emergency scenarios as emergencies warranting ambulance attendance. For example, a woman going into labour has often been miscategorised.^13^, ^16^ Although data from the present study suggested men were more likely to perceive routine labour as an ‘emergency’ warranting a call for an emergency ambulance compared to women, the difference was only small (52% *vs* 48%, respectively, *P* < 0.001). Scenario wording expressed no reason to suspect labour complications, suggesting that even for normally progressing labours, a substantial proportion of the public's first notion would be to call for an ambulance.

Overall, our data suggests women are less likely to call for an ambulance for non‐emergency scenarios. This finding seems contentious in consideration of corresponding literature, suggesting either women are more likely to call for an ambulance^17^ or attend EDs^18^, ^19^ for non‐emergency medical situations, no differences between men and women^20^, ^21^ or that (as was the case with our data) men are more likely to utilise EHS for low acuity conditions.^22^ Findings likely differ across the literature given the range of methods used to identify appropriate *vs* inappropriate EHS use. The majority of previous investigations focus on actual EHS users, as opposed to hypothetical EHS use among the general populace. One other UK study providing hypothetical scenarios to general population participants (as opposed to EHS users specifically) found no differences for inappropriate ambulance use between males and females.^16^ Further research – both among the general population (who can call for an emergency ambulance at any time) and retrospective analysis of actual emergency ambulance users – may be warranted to definitively ascertain between‐sex differences.

Our data also suggested people aged 56+ years were more likely to suggest non‐emergency scenarios warranted a call for an emergency ambulance than those aged 18–35 years. This is counter to the majority of pre‐existing literature suggesting either young people are more likely to inappropriately engage with EHS^18^, ^23^ or little to no differences across different age groups.^20^ Previous research suggests younger people are more likely to directly seek EHS attention, often because of the added convenience EHS offers over other non‐emergency healthcare pathways.^18^, ^24^ Interestingly, another study presenting hypothetical non‐emergency scenarios also found older people more likely to call for an emergency ambulance than younger people.^25^

We noted those who had completed a university degree and had higher household income were less likely to call an ambulance for non‐emergency scenarios (previous research also links heightened education with more appropriate ambulance use,^26^ as were people with higher emotional wellbeing as measured via the BEES. Although several studies have investigated the impact of patients utilising EHS for acute mental health issues, few have investigated the association between poor mental and/or emotional wellbeing and potentially unnecessary use of EHS. One study found heighted ambulance use for minor conditions among those with a psychiatric disorder,^27^ with another review article suggesting some interventions targeting social/emotional issues among patients can reduce unnecessary EHS use.^28^

### Implications

Patients may choose to access EHS where it may not be entirely necessary because of limited confidence in other healthcare pathways, convenience, perceived urgency of their condition, or a perception their condition may require resources and/or facilities not available through other healthcare pathways.^24^ Undoubtedly, there are other aspects outside of an individual's knowledge of what should and should not warrant EHS engagement that contribute to the decision of which healthcare pathway should be taken. For example, a perception of not being able to obtain a timely appointment with ones GP can lead to increased EHS engagement. More globally, although wider‐reaching public health interventions addressing poverty, homelessness and support for childcare will reduce impact on EHS, undoubtedly consideration of increased system capacity in alignment with increased demand is key.^7^ Nonetheless, to address knowledge at the individual level which may have some small capacity to alleviate system pressures, educational initiatives targeting reducing delay seeking help when needed, services provided by GPs, ambulance services and EDs, and guidance about the clinical urgency of symptoms (and the most appropriate healthcare pathway for managing these) are suggested.^29^

### Strengths and limitations

Inherent strengths of this present study include: (1) our ability to leverage trialled study materials (including medical scenarios) from a similar previously published investigation, (2) the additional incorporation of graphics alongside text to improve contextualisation of medical scenarios, and (3) the representative nature of the Australian adult population from which data was derived.

However, this present study is not without limitations. For example, participants were recruited via an online market research company which did include some small incentive for participation. Although self‐selection bias is unlikely to have impacted in any meaningful way on study results, we do acknowledge the non‐random nature of the sampling frame. Further, it should be noted that, even with graphic images aiding contextualisation of textual medical scenarios, it is conceivable individuals could interpret scenarios in different ways. Scenarios utilised focussed on clinical information pertaining to primary health concerns but did not consider other potentially relevant aspects such as comorbidities or social issues. Future research focussing on factors that contribute towards decision‐making in emergency ambulance utilisation would be of benefit.

Data collection occurred in November/December 2020. Although national COVID‐19 infection rates were (comparatively) low during this period, data was collected in the midst of a global pandemic, whereby it has been suggested people have been less willing to engage with EHS out of fear of exposure to SARS‐CoV‐2.^30^ It is unclear the extent to which attitudes changing in retaliation of the COVID‐19 pandemic may have impacted on study findings.

Last, defining unnecessary ambulance use is complex and often subjective. For example, it can sometimes be necessary for paramedics to transport patients to hospital EDs for non‐clinical reasons. ‘Unnecessary’ use is not always a deliberate misuse, particularly as research suggests many individuals are hesitant to engage with EHS.^29^ Although our classification of non‐emergency scenarios not warranting an emergency ambulance response came from a panel of experienced paramedic perspectives based on information present in medical scenarios, it is acknowledged binary judgement of appropriateness of emergency ambulance engagement lacks nuance and consideration of some individual circumstances.

## Conclusions

Emergency ambulance use for low acuity conditions continues to contribute to the stretched service capacity of healthcare systems around the world. Until service capacity and integrated healthcare pathways are improved, enhancing understanding among the general public of the situations that warrant emergency ambulance intervention (and those that do not) will play a small role in easing burden on jurisdictional ambulance services, EDs and their staff.

## Supporting information


**Table S1.** Emergency and non‐emergency scenario text with graphics.

## Data Availability

Derived data supporting the findings of this study are available from the corresponding author on request.
