# Supplementary material for: Calling an ambulance for non‐emergency medical situations: Results of a cross‐sectional online survey from an Australian nationally representative sample
Source: Emerg Med Australas. 2022 Sep 16;35(1):133–41. doi: 10.1111/1742-6723.14086 (PMC10087376; doi:10.1111/1742-6723.14086)
Supplement: Supplementary file 1 — Table S1. Emergency and non‐emergency scenario text with graphics. [file EMM-35-133-s001.docx]

Supplementary Material Table 1. Emergency and non-emergency scenario text with graphics

|  | Scenario number | Short scenario Title | Full scenario text | Scenario graphics |
| --- | --- | --- | --- | --- |
| Emergency Scenarios | 1 | Box Jellyfish sting | Whilst in Northern Queensland, a boy is stung by a Jellyfish while swimming at the beach, and large welts appear on his arm. | 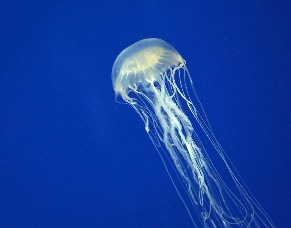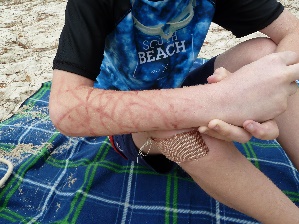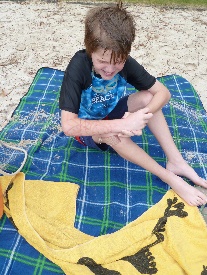 |
|  | 3 | Snake bite (unidentified) | A 50-year-old woman has been bitten on her ankle by an unidentifiable snake. | 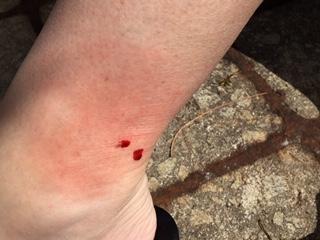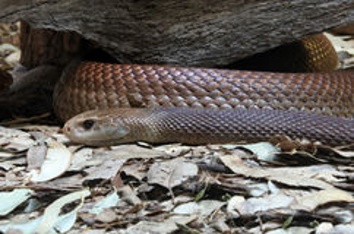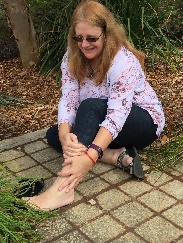 |
|  | 5 | Mild chest pain | A 40-year-old woman is experiencing mild chest pain. She does not think it is indigestion or a strained muscle. | 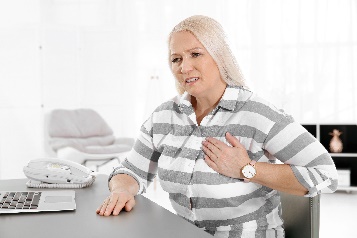 |
|  | 8 | Stroke | A 67-year-old man is slurring his words; he has not drunk any alcohol. | 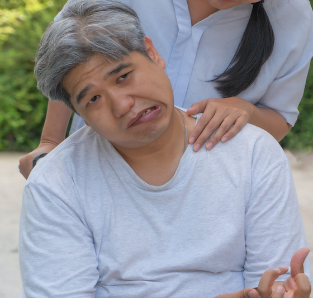 |
|  | 9 | Severe chest pain | A 52-year-old man has severe chest pain, is sweating and grey in colour. | 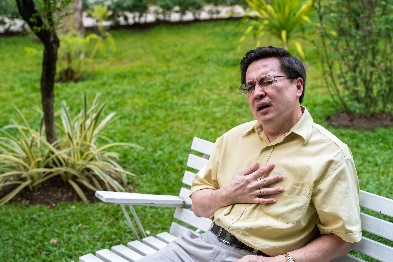 |
|  | 13 | Paracetamol overdose | A 32-year-old female has taken 10 regular paracetamol tablets in the last 12 hours, and is feeling extremely unwell. She has abdominal pain and feels nauseous. | 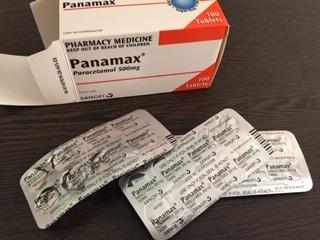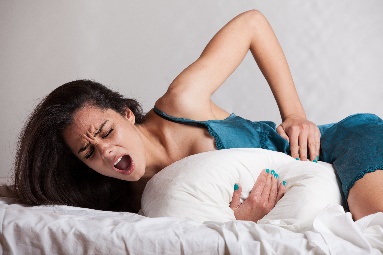 |
|  | 15 | Child head haematoma | A 3-year-old boy has fallen off the couch and bumped his head. He began crying immediately and a golf-ball size lump with a bruise promptly appears. | 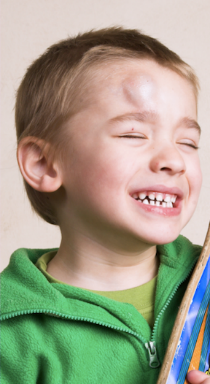 |
|  | 16 | Potential meningococcal disease | A 4-year-old girl has woken up with a high temperature, feels hot to touch, has a really sore neck and a headache which Panadol is not relieving. | 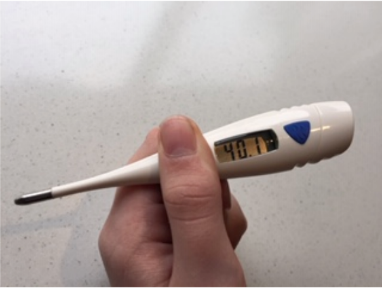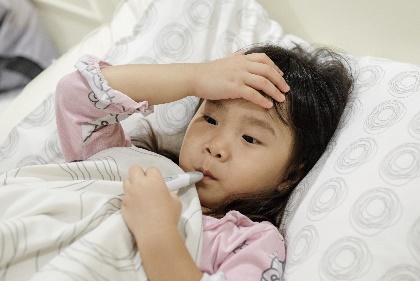 |
|  | 17 | Older person hip pain | An 80-year-old woman feel out of bed, is now unable to get up and is complaining of hip pain on her right side. | 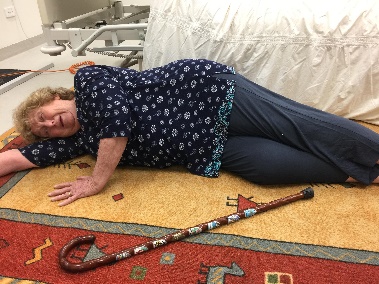 |
| Non-emergency scenarios | 2 | Flu | A 45-year-old male has flu-like symptoms. He has a mild fever, cough, headache, runny nose and feels tired. | 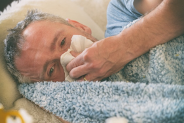 |
|  | 4 | Older person bruising | A 77-year-old woman knocks herself against the kitchen table, and a large bruise immediately appears on her thigh. | 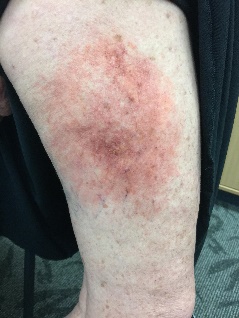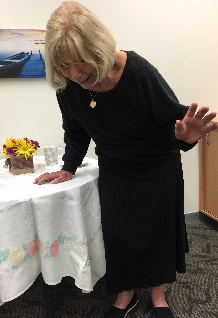 |
|  | 6 | Lego in ear canal | A 4-year-old girl has a Lego piece stuck in her ear canal. | 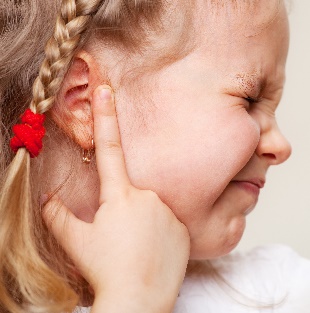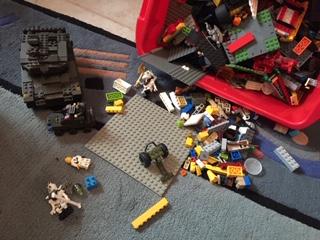 |
|  | 7 | Stubbed toe | A 25-year-old male is playing football with his friends in his backyard with his bare feet. He stubs his toe on a brick. There is blood and he suggests it is throbbing quite painfully. | 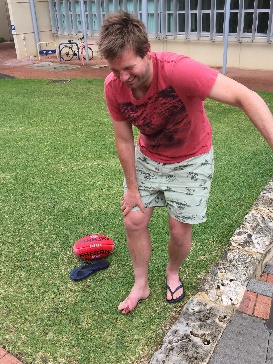 |
|  | 10 | Alcohol intoxication | A 22-year-old male is conscious, not injured and has drunk a substantial amount of alcohol on a night out. | 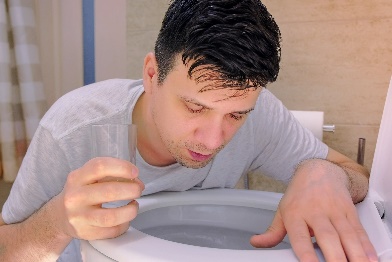 |
|  | 11 | Woman in labour | A 33-year-old woman is 9 months pregnant and goes into early stages of labour. Her waters have broken, and she feels uncomfortable. | 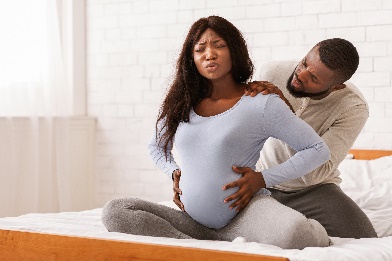 |
|  | 12 | Back pain | A 40-year-old man with a 6-month history of back pain wakes up in the middle of the night with a sore back and has run out of pain killers. The man is in quite a bit of pain. | 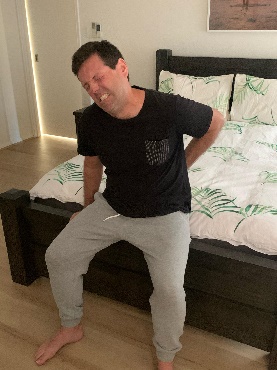 |
|  | 14 | Cut finger | A 42-year-old man has cut his finger while chopping vegetables, and the bleeding is controlled with pressure. | 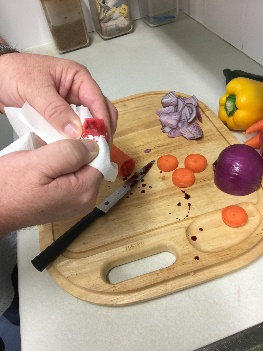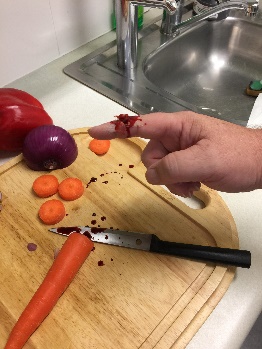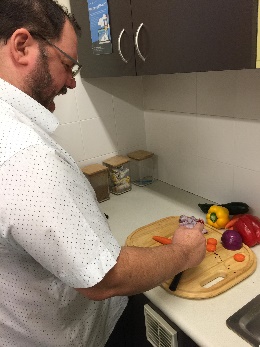 |
